# Assessing the impact of energy and fuel poverty on health: a European scoping review

**DOI:** 10.1093/eurpub/ckad108

**Published:** 2023-07-12

**Authors:** Sarah N Champagne, Euan Phimister, Jennie I Macdiarmid, Aravinda Meera Guntupalli

**Affiliations:** Institute of Applied Health Sciences, School of Medicine, Medical Sciences and Nutrition, University of Aberdeen, Aberdeen, UK; Department of Economics, Business School, University of Aberdeen, Aberdeen, UK; Stellenbosch Business School, Stellenbosch University, South Africa; Rowett Institute, School of Medicine, Medical Sciences and Nutrition, University of Aberdeen, Aberdeen, UK; Institute of Applied Health Sciences, School of Medicine, Medical Sciences and Nutrition, University of Aberdeen, Aberdeen, UK

## Abstract

**Background:**

The burden of energy and fuel poverty (EFP) in Europe is increasing in the face of the cost-of-living crisis, the Russian invasion of Ukraine, the coronavirus disease 2019 (COVID-19) pandemic and the climate emergency. While the health impacts of EFP are often the driving reason for addressing it, EFP’s association with health is poorly delineated. This review aims to scope the evidence of EFP’s association with health in Europe.

**Methods:**

A scoping review based on Arksey and O’Malley’s framework was conducted using search terms relevant to EFP, health and Europe. Five databases were searched, in addition to hand searching. Review selection was performed by two independent reviewers, and articles were thematically analyzed.

**Results:**

Thirty-five articles published between January 2000 and March 2022 were included. The literature varied in definitions and measurements of EFP and in the health indicators examined. The review revealed a negative association between EFP and health, specifically, general unspecified poor health (9 articles), excess winter mortality (3 articles), communicable diseases (3 articles), non-communicable diseases (11 articles), mental health (15 articles) and well-being (12 articles). While women were reported to be at a higher risk of EFP than men, children and older adults were identified as particularly vulnerable to EFP’s adverse health repercussions.

**Conclusions:**

This scoping review illustrates a significant and complex association between EFP and various domains of health. Though heterogeneity across research makes it difficult to compare findings, our review supports the use of health as a justification to address EFP and urges public health to be more involved in EFP mitigation.

## Introduction

While energy poverty (EP)—or fuel poverty (FP)—is a long-standing issue, Europe is facing an unprecedented energy and fuel poverty (EFP) crisis due to the ongoing cost-of-living crisis and the Russian invasion of Ukraine that are driving higher fuel prices amidst the backdrop of the ever-present coronavirus disease 2019 (COVID-19) pandemic and climate emergency. A household is defined as energy- or fuel-poor if they are unable to use household appliances and adequately heat and/or cool one’s home to maintain decent living standards.^1^ EFP may be driven by a lack of resources, an inability to access fuel sources, housing inefficiencies, above-average energy needs or a combination thereof.^2^^,^^3^ Exposure to EFP could hence, for example, result in cold, damp and mouldy dwellings and limit people’s abilities to prepare warm meals or take hot showers. Such exposures may contribute to poor health and well-being.^4^^,^^5^

In 2009, the European Fuel Poverty and Energy Efficiency project estimated between 50 and 125 million people experience poverty across Europe.^6^ While health is often used as a justification to address EFP, EFP definitions, measurements and corresponding health consequences are often varied and vaguely detailed.^2^^,^^7^ EFP’s impact on health is likely complex and has many potential covariates that impact health outcomes, making it difficult to measure and disentangle, especially if relevant covariates are excluded from the study. This may explain why EFP has been condensed with all cold-related illnesses or has been assessed in tandem with housing poverty.^1^^,^^8^ Despite the numerous associations with health, EFP seems to often rest within the financial and economic disciplines, instead of public health.^2^^,^^9^ The aim of the study, therefore, is to scope the existing European evidence of the association specifically between EFP and health, inclusive of well-being.

While some existing studies have summarized a portion of the existing literature on the impact of the EFP on health, no systematic synthesis has been carried out.^7^^,^^8^^,^^10^^,^^11^ Moreover, of these few reviews, some focused exclusively on intervention methods and most did not centre health as the core outcome. Therefore, the primary purpose of the study is to conduct a scoping review of the association between EFP and health and well-being, identifying key concepts, definitions and gaps in research. The secondary purpose is to map differences in EFP and health associations among vulnerable groups.

## Methods

This scoping review was conducted based on Arksey and O’Malley’s methodological framework of scoping studies.^12^ The framework maintains components of systematic reviews—e.g. that the review should be rigorous and transparent—while allowing for a more iterative process with a broader research focus.^12^

Three concepts were included in the search strategy, each with their respective related search terms: EFP, health (inclusive of physical health, mental health, and well-being) and Europe (studies specific to European countries). For more information on the search strategy, see Supplementary Appendix S1.

Five electronic databases—Medline, Embase, Web of Science, Sociological Abstract and EconLit—were searched in April 2022. To identify additional academic and grey literature, hand-searching and searching through publications’ reference lists were conducted. English language articles published between 2000 and March 2022 were included in the search.

**Figure 1:**
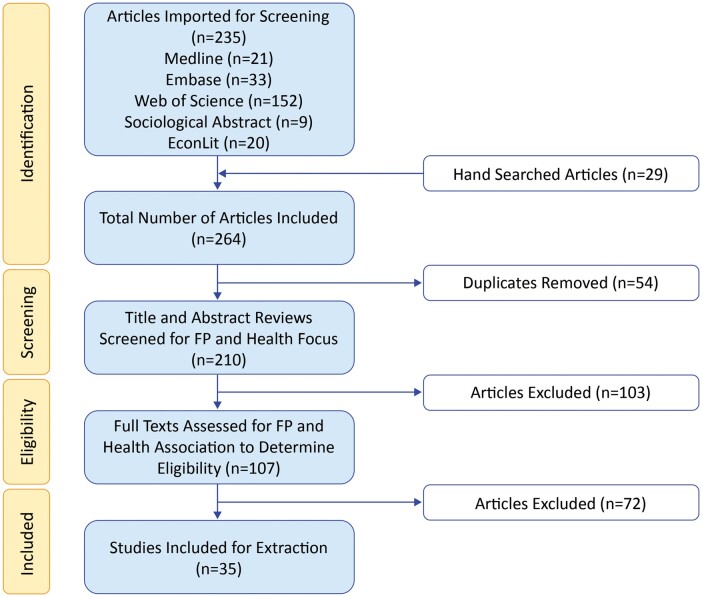
PRISMA flowchart of study selection process

Two researchers independently conducted title and abstract screening, and full-text analysis. In cases of conflicting eligibility, a third researcher was consulted to reach a consensus. Studies that specifically looked at the *association* between EFP and health and/or well-being met the inclusion criteria of this review. Studies that focused broadly on overarching poverty, income or monetary poverty, or on housing poverty, without speaking specifically to energy or fuel deprivations were excluded. Furthermore, books, literature reviews and studies published after 2000 that explored only secondary data from the 1900s were excluded. Consistent with Arksey and O’Malley’s methodological framework, no quality criteria were included. For details on the search and selection process, see figure 1.

Data extraction points were identified inductively to address the aims of the review. The following data were extracted: title, authors, date of publication, journal of publication, type(s) of methods used, description of EFP interventions (if relevant), location, population, aims, methodology and data sources, controls, and disaggregation, EFP definition and measurement, health definition and measurement, physical or general health associations, mental health associations, other dimensions of poverty discussed and limitations. The extracted data were then thematically analyzed.^12^ Themes emerging from articles’ definitions or understandings of EFP were later grouped into corresponding ‘elements.’ Ten definitional elements were inductively identified and are detailed below.

## Results

The scoping review yielded 35 studies. A complete list of the studies with the year of publication, methodology, EFP measure and health measure can be found in Supplementary Appendix S2.

The results are organized as follows: (i) description of the articles included, (ii) presentation of the thematic analysis of the definitions of EFP, (iii) delineation of the health associations with EFP identified and (iv) demographical differences among EFP health associations.

As table 1 illustrates, most articles were published after 2015. Articles specific to the UK were overrepresented within the literature. Five papers focused on pan-European EFP and health associations, whereas 16 were national, 6 were sub-national and 9 were local in scope. Twelve articles examined the impact of EFP interventions on health outcomes as opposed to the association between EFP and health and well-being broadly. For further details, see table 1.

**Table 1:** Article characteristics

Article characteristics	*N*	(%)	Relevant citations
Date of publication			
2000–04	1	3	57
2005–09	2	6	37, 40
2010–14	4	11	26, 36, 48, 51
2015–19	16	46	11, 16, 17, 22, 24, 25, 32, 33, 39–45, 49, 61
2020–22	12	34	3, 5,18–20, 23, 30, 31, 35, 38, 62, 63
Journal sector			
Energy and Economics	12	34	16, 22, 23, 25, 31, 32, 38, 41, 49, 51, 61, 62
Public Health and Medicine	16	46	3, 4, 19, 20, 24, 30, 33, 36, 37, 39, 40, 42, 44, 48, 57, 63
Space and Housing	5	14	5, 17, 18, 26, 45
Grey literature/other	2	6	35, 43
Study location			
Europe	4	11	4, 17, 39, 41
Belgium	2	6	22, 35
France	1	3	24
Greece	1	3	16
Ireland	4	11	38, 43, 57, 63
Spain	6	17	3,18–20, 23, 62
UK	17	49	5, 25, 26,30–33, 36, 37, 40, 42, 44, 45, 48, 49, 51, 61
Methodology			
Qualitative	5	14	23, 25, 32, 35, 37
Mixed-methods	1	3	30
Cross-sectional	18	51	3–5,17–20, 22, 24, 31, 33, 36, 39, 41, 43, 48, 51, 57
Cohort study	2	6	38, 63
(Quasi) Experimental	2	6	26, 42
Longitudinal	3	8	16, 40, 62
Ecological	1	3	44
Spatial analysis	1	3	45
Other	2	6	49, 61
Presence of a EFP intervention			
Yes	12	34	3, 23, 26, 30, 33, 37, 40, 42, 44, 49, 51, 61
No	23	66	4, 5, 16,17,18,19,20, 22, 24, 25, 31, 32, 35, 36, 38, 39, 41, 43, 45, 48, 57, 62, 63
Terminology used for EFP			
Energy poverty	12	34	3, 4, 19, 20, 22, 23, 35, 38, 41, 45, 51, 63
Fuel poverty	23	66	5,16–18,24–26,30–33, 36, 37, 39, 40, 42–44, 48, 49, 57, 61, 62

### Key concepts and definitions of EFP

Within the literature, the term ‘fuel poverty’ was found to be used across European countries; however, among continental European publications, the term ‘energy poverty’ was the more common and is the term used by the European Commission.^13^ In low- and middle-income countries, EP and FP may be used to describe slightly different phenomena, in high-income countries the terms are largely synonymous.^14^^,^^15^ To emphasize our inclusion of both EP and FP, we rely on the acronym EFP.

Twenty-nine out of 35 European EFP studies focused exclusively on cold weather EFP, whereas six articles explicitly included the inability to cool or maintain a comfortable household temperature during the warmer months in their definition of EFP.^4^^,^^16^^–^^20^ Of the studies that did include it in their definition, four studies (from warmer countries) measured or included findings specific to the health associations of an inability to cool. The correlated health associations of overly warm households included excess summer mortality and cardiovascular and respiratory diseases.^16^^,^^19^

Beyond the inclusion of cooling, EFP definitions and measurements varied greatly. Many articles’ definitions of EFP included several elements. As table 2 highlights, most articles articulated a lack of ability to keep the household adequately warm as a tenet of EFP. Of those articles, most rely on self-reported (in)adequacy of warmth, but some provided exact temperatures—21C in the main room and 18C in subsequent rooms, as recommended by the World Health Organization.^21^ Likewise, 12 articles’ definitions included the inability to meet basic energy supply needs or to access hot water, cooking appliances, lighting and other electric or gas needs. Sixteen EFP definitions focused on individual or household income in relation to fuel prices or expenditure.^49^ For more details, see table 2.

**Table 2:** Elements of EFP’s definition

Elements of EFP	***N*** ^a^	Relevant citations
Lack of ability to keep households adequately warm based on self-reported assessment	26	3–4,16–20, 22, 24,31–33, 35, 36, 39–43, 45, 48, 51, 57, 62, 63
Lack of ability to keep houses adequately warm based on the WHO recommendations (21C in the main room, 18C in subsequential occupied rooms)	4	5, 19, 24, 48
Lack of ability to keep households adequately cool based on self-reported measures	6	4,16–20
High cost of fuel, low income	7	23, 30, 31, 38, 39, 44, 57
Paying more than 10%, or an excessive part of income on heating	10	25, 26, 31, 36, 37, 39, 40, 43, 48, 49
Difficulty, delays or arrears in payment of energy bills	8	3,16–18, 22, 42, 51, 63
Lack of access to energy sources	4	16, 19, 22, 45
Inability to meet basic energy supply needs	6	3, 16, 17, 23, 31, 35
Inability to afford to use household appliances and utilities	7	17, 19, 23, 24, 39, 45, 62
Inability to quell dampness and mould	6	3,16–18, 36, 51
No definition provided	1	61

a Many articles included several elements in their definitions and hence are represented multiple times here.

Measurement correspondingly differed depending on the definition of EFP and, presumably, data availability. Some studies measured individuals who received an intervention, even when the inclusion criteria for the given intervention were not based on EFP status. Most articles relied on a self-reported inability to keep adequately warm.

### Key findings on the association between EFP and health

While all articles cited an association between EFP and health, the intervention-based studies focused exclusively on health outcome measures (e.g. change in the measured health status of individuals or groups following an intervention), and the remaining quantitative and qualitative studies focused on health in a cross-sectional or longitudinal manner or on the mere association between EFP and health. As table 3 illustrates, most included studies suggested a particularly prominent association between poor mental health and non-communicable diseases (particularly respiratory disease).

**Table 3:** Primary and secondary analyses of health associations between EFP and health and well-being

Health association with EFP	*N*	Relevant citations
Unspecified general poor health	9	4, 19, 20, 30, 31, 35, 37, 45, 62
Excess winter mortality	3	5, 16, 44
Non-communicable diseases	11	3, 5, 19, 20,30–33,35–37
Cardiovascular disease	3	19, 31, 33
Circulatory disease	2	32, 33
Respiratory disease	10	3, 5, 19, 20, 30, 32, 33,35–37
Arthritis and inflammation	2	30, 31
Injuries and falls	1	19
Communicable diseases	3	30, 33, 37
Cold	3	30, 33, 37
Flu	1	33
Mental health	15	3–5, 19, 20, 22, 25,30–33, 37, 45, 51, 63
Depression	4	3, 4, 25, 37
Anxiety	4	3, 25, 30, 37
Difficulty managing emotions	5	20, 22, 25, 30, 51
General poor mental health	10	5, 19, 20, 25,30–33, 45, 63
Well-being	12	4, 5, 20, 25,30–33, 35, 40, 41, 42

#### Unspecified general poor health

Looking at the association between EFP and health broadly, people who experienced EFP felt they had less control over their health,^22^ spent more money on health-related quality of life costs^23^ and reported poorer self-assessed health.^17^^,^^19^^,^^23^^,^^24^ In a qualitative study by Mould and Baker, negative health impacts were shown to last even once a household is lifted out of EFP.^25^ Moreover, a randomized control trial study by Heyman et al. suggested that EFP interventions do not always result in a change to self-reported health status.^26^ This may be influenced by the types of EFP interventions, such as those focusing on household energy efficiency, or the duration of time post-intervention measured.

#### Excess winter mortality

Excess winter mortality (EWM) is the difference between the number of actual deaths in the winter months compared with the expected number of deaths and is associated with cold strain from indoor and outdoor environments.^27–29^ However, many European countries with comparatively milder winters experience higher rates of EWM.^4^^,^^16^^,^^30^ EFP has been shown to increase EWM and may partially explain this anomaly.^5^^,^^16^ Atsalis et al., in a longitudinal study, identified a positive correlation between mortality rates and households with an inability to keep adequately warm in the winter months in Greece which could account for 1080–2962 deaths per year.^16^

#### Non-communicable diseases

Rates of cardiovascular disease rise as temperatures decrease in the winter months and have been shown to increase even more among households in EFP who cannot keep the household adequately warm.^16^^,^^31^^–^^33^ As highlighted by Chard and Walker in a qualitative study, this correlation is particularly acute among older adults.^32^ Circulatory disease and cerebrovascular disease are similarly affected by lower temperatures and EFP.^32^^,^^33^ Continued exposure to cold temperatures narrows blood vessels and increases the blood’s viscosity, raising blood pressure and increasing the risk of stroke and heart attack.^34^

Respiratory disease and problems are strongly associated with EFP.^3^^,^^5^^,^^19^^,^^20^^,^^30^^,^^32^^,^^33^^,^^35^^–^^37^ Prolonged exposure to cold air can increase broncho-construction and the presence of mucus and damp-mouldy dwellings can trigger allergic reactions and lower resistance to respiratory infections, collectively increasing the risk of respiratory disease.^38^

While authors often did not distinguish between causality and aggravation of existing symptoms, arthritis,^30^ chronic inflammation^31^ and increased risk of falls and fractures^19^ are subsequent health conditions that have been shown to be associated with EFP in the literature.

#### Communicable diseases

The common cold and influenza (flu), two communicable diseases often associated with the winter months, also seem to be intensified by EFP.^30^^,^^33^^,^^37^ Exposure to cold air may weaken resistance to these respiratory illnesses.^34^ A fifth of participants in a qualitative study by Gilbertson et al. self-reported fewer instances of cold and flu following a UK EFP intervention known as the Warm Front Scheme.^37^

#### Mental health

The existing evidence suggests that the association between EFP and mental health is particularly acute.^31^ Among mental health conditions, EFP is specifically associated with depression,^3^^,^^4^^,^^25^^,^^37^ anxiety^3^^,^^25^^,^^30^^,^^37^ and difficulty managing emotions.^20^^,^^22^^,^^25^^,^^30^^,51^ Worry and stress surrounding affording fuel and fuel-related debts (feelings of a lack of control), as well as the discomfort of being physically cold, likely contribute to poor mental health.^25^^,^^30^^,^^32^^,^^37^ Furthermore, a qualitative study by Mould and Baker reported that people who experience EFP report higher rates of isolation, a known risk factor for mental health.^25^ The association with isolation may be due to the desire to host fewer visitors in a cold home. Mental health may also have a causative impact on EFP, as debt and mental health are mutually reinforcing.^25^

#### Well-being

Related and intertwined with mental and physical functioning, well-being is also associated with EFP. People experiencing EFP tend to have lower self-reported well-being.^4^^,^^5^^,^^20^^,^^25^^,^^30^^–^^33^^,^^35^^,^^39^^,^^41^ A quasi-experimental study by Grey et al. suggested that EFP interventions show promise in increasing subjective well-being among the previously energy poor.^42^

### Differences in demographics

Only six articles disaggregated findings by age group providing insights into the different health impacts of EFP over the life course.^20^^,^^36^^,^^38^^,^^40^^,^^43^^,^^44^ Those that did highlighted a pronounced association between children experiencing EFP and respiratory disease (such as asthma and bronchitis), being overweight and facing peer and emotional problems.^20^^,^^38^ In a cross-sectional study by Oliveras et al., children and adolescents experiencing EFP were associated with poor mental health and expressed feeling unhappy with their families, poorly cared for and afraid of being bullied.^20^ Moreover, the association between EFP and EWM, cardiovascular disease, depressive symptoms and respiratory disease appears to be stronger among older adults compared with other adults.^36^^,^^40^^,^^43^^,^^44^ Murage et al., in an ecological study, reported older adults experiencing EFP have the highest mortality risk at 75 years of age and above.^44^ Vulnerability to EFP health consequences may be due to older adults often having less subcutaneous fat than younger adults, weakening their temperature control and being more likely to experience pre-existing conditions.^39^^,^^40^

Beyond age, five studies disaggregated data to examine how health and EFP dynamics differently impact women compared with men, and five more controlled or adjusted for demographics, such as gender. The gender disaggregated research revealed that women are more vulnerable to EFP, thus widening health inequalities.^3^^,^^19^^,^^45^ Gender roles expose women to longer periods of time in the household compared with men.^3^^,^^45^ Women, despite having higher life expectancies, tend to have higher rates of disability and illness which may make them more vulnerable to potential EFP health impacts.^19^^,^^45^ Furthermore, women facing EFP seemed to experience poorer mental health and self-reported health compared with men.^39^ Receptively, in a cross-sectional study by Marí-Dell’Olmo et al., an EFP intervention showed to be more protective to women’s health than men’s.^39^ The review identified no information about gender minorities. One cross-sectional study by Carrere et al. examined how immigrant status interacts with health and EFP, noting that immigrants may be more vulnerable to EFP than non-immigrants.^3^ No study disaggregated for rural/urban status nor for household composition.

## Discussion

This review identified 35 articles that suggested a potential relationship between EFP and health. Thirty-four percent of studies explored the health outcomes and/or associations of EFP interventions and 51% of studies were cross-sectional in design. There was no clear link between the methods selected and the health indicators explored. Both mental and physical health, including non-communicable diseases, communicable diseases and EWM, are shown to be negatively associated with EFP.^3^^,^^5^^,^^13^^,^^19^^,^^27^^,^^33^^,^^36^^,^^38^^,^^42^^,^^43^^,^^45^ Despite being popular academic topics independently, EFP and health jointly contributed to relatively little academic discussion and debate.^46^ However, in recent years, we have seen a surge in interest in the topic, as evidenced by 28 of the 35 included articles being published in the later 7 years of the review. More attention is required given the scale and the scope of the impact of EFP on health in Europe.

Consistent with scoping review methodology, our analysis did not formally incorporate a quality appraisal. However, we did observe heterogeneity within the research impacting quality and making it difficult to compare findings. For one, the concept of EFP has many definitions. Definitional variations impact *what* is being studied. For instance, the health consequences of living with fuel debts or not being able to cook a hot meal may be different from those of residing in cold dwellings. Disentangling which elements of EFP contribute to a given health consequence is important to explore and to better design EFP health interventions. Furthermore, the scoping review identified a lack of focus on summer and/or cooling EFP. The limited evidence suggests a negative association between summer EFP and health.^28^^,^^42^ Nevertheless, the paucity of research came as a surprise given the 70 000 excess summer deaths in Europe during the summer heatwave of 2003.^47^ The lack of focus on cooling may be impacted by the over-representation of the UK and Ireland (relatively colder climate European countries) in the literature. Nevertheless, the increase in extreme heat events due to climate change calls for the definitional inclusion of cooling within EFP and additional European summer EFP research.

Often, EFP measures rely on a complex conceptualization of expenditure that influences ***who*** is being studied. High-expenditure households (those spending more than 10% of their income on fuel) may be considered as one entity despite their variability of income. As such, those that have large households and hence require more heating but can afford to spend a higher percentage of their income on heating are grouped with those who cannot afford to heat their modest households. Moreover, proxy measurements of EFP were used in some studies further influencing the populations of interest. For example, some research studied all those who received an intervention or relied on vulnerability to EFP as a substitute for the presence of EFP.^30^^,^^42^^,^^49^ These tactics may exclude people who would fit into the paper’s definition of EFP or include people who do not, impacting health associations. Moreover, additional research is needed to purposefully explore the lived experience of EFP, specifically among vulnerable groups. For instance, older adults experience particularly high morbidity and mortality risks that require additional exploration.^40^

The review clearly illustrated that the EFP and health associations are complex and multifaceted. This may be for two primary reasons. First, EFP is embedded more widely in multidimensional poverty. Within the field of multidimensional poverty—which, unlike income poverty alone, looks to reveal both *who* is poor and *how* they are poor, acknowledging that people may experience multiple deprivations at a time^50^—this literature review exemplifies both the importance of studying unique dimensions of poverty in the form of EFP and the complexity and intersection of EFP with various dimensions of poverty. For instance, EFP and housing poverty are often overlapping and frequently experience and explore together.^36^^,^^39^^,^^43^^,^^51^ EFP is also embedded in the European Union material deprivation measures.^52^ Similarly, EFP may impact peoples’ ability to cook warm meals which intersects with food poverty and may contribute to unique health consequences, as well as the understudied ‘heat or eat’ trade-off described in news media.^42^^,^^53^

A second potential reason for the complex association between EFP and health may be that health indicators could be impacted by socioeconomic indicators other than EFP poverty. For example, potential cramped households or precarious and/or low-pay employment, among people experiencing EFP, could impact the health indicators measured in EFP studies despite not being present in the analysis.^54–56^ Despite EFP’s entanglement with multidimensional poverty and other socioeconomic indicators, the variety and severity of EFP’s negative health associations strongly suggest that EFP is an important deprivation and still necessitates targeting. As such, we argue that EFP should be addressed both at the macro-level (within multidimensional poverty) as well as at the micro-level exploring specifically EFP.

Only five articles in our review included dialogue with the health system and none had discourse with healthcare professionals.^19^^,^^23^^,^^30^^,^^33^^,^^51^ While a small number of studies touched on the correlation between healthcare uptake or expenditure and EFP, such as Oliveras et al.’s examination of healthcare utilization among people experiencing EFP, the health system was overall not identified as a milieu to flag or assist people experiencing EFP.^19^^,^^23^^,^^33^^,^^51^ This may be because EFP is insufficiently framed as a public health issue as opposed to an economic issue, despite its impact on health. More ties between the health system and EFP should be explored and established to mitigate potential health consequences.

Despite the rigorous methods employed by the team, guided by Arksey and O’Malley’s framework, the review may not have identified all published accounts of the EFP and health, particularly as research prior to 2000 and books were not included.^12^ Though related, works specific to energy justice or housing poverty that did not include EFP markers were also excluded. Furthermore, only English language literature was included in the review, which may contribute to the overrepresentation of English-language countries (the UK and Ireland) in the studies reviewed. This overrepresentation may be further driven by the poor housing tenure and relatively high burdens of EWM in the UK and Ireland compared with much colder continental European countries.^4^ Additional research exploring the association between EFP and health is needed in continental Europe, especially with the increase in anecdotal reports of people (particularly from Eastern Europe) burning illegal and/or toxic materials to stay warm amidst the energy crisis.^58–60^

While numerous studies noted associations between EFP and facets of health, the prominence of the association may be a result of research questions targeting some health associations and neglecting others.^3^^,^^25^^,^^30^^,^^37^ Hence, while the health associations detailed in this review highlight the importance of addressing EFP, this may not be an exhaustive account. Our review also highlights the need for detailed research on the health impacts of EFP by disaggregating the impacts by age, gender and other socio-economic characteristics. Given the increasing impact of the cost-of-living crisis and the ongoing Russian invasion of Ukraine, we believe that EFP must be studied and addressed more rigorously and through a health lens in Europe.

## Conclusion

Our study is the first review to analyze the existing literature on the association between EFP and health in Europe. Complementarily to existing literature, the evidence illustrates a significant and complex association between EFP and various domains of health. The most prominently researched health impacts range from respiratory disease to poor mental health and hampered well-being.^3^^–^^5^^,^^20^^,^^30^ Older adults and children are reportedly particularly vulnerable to the negative health consequences of EFP compared with adults.^20^^,^^36^^,^^38^^,^^40^^,^^43^^,^^44^ Early research moreover suggests that women are increasingly vulnerable to EFP and may hence face disproportionate health consequences, compared with men.^3^^,^^19^^,^^39^^,^^45^ Given the multidisciplinary nature of this area of research, the use of comparable and specific definitions and measures of EFP is needed, instead of relying on a broad concept like a ‘cold home’. More in-depth research is also needed. Particularly, research on health and the inability to cool, the lived experiences of people in EFP, EFP among hard-to-reach and vulnerable groups, and the impact of the energy crisis on EFP in continental Europe. This research could help strengthen EFP policy within Europe, which is distinctly important in today’s socio-political climate. This scoping review supports the use of health as a catalyst to address EFP; however, it stresses the importance of further engaging health systems, health professionals and public health broadly as a field in working to confront EFP.

## Supplementary Material

ckad108_Supplementary_Data

## Data Availability

The data underlying this article are available in the article and in its online supplementary material. Energy and fuel poverty (EFP) has a myriad of definitions, indicators and measurements impacting both who and what is being studied, making it difficult to compare findings. This scoping review revealed a significant association between EFP and various domains of health, namely poor mental health (such as anxiety and depression) and physical health, including non-communicable disease (such as cardiovascular and respiratory diseases), communicable disease (such as cold and flu) and excess winter mortality. Women are more vulnerable to experience EFP compared with men, and children and older people are more strongly impacted by the health consequences of EFP compared with adults. We recommend health systems, health professionals and public health broadly as a field be more involved in working to confront EFP.
