# Correction to: Assessing the impact of energy and fuel poverty on health: a European scoping review

**DOI:** 10.1093/eurpub/ckad166

**Published:** 2023-09-20

This is a correction to: Sarah N Champagne, Euan Phimister, Jennie I Macdiarmid, Aravinda Meera Guntupalli, Assessing the impact of energy and fuel poverty on health: a European scoping review, *European Journal of Public Health*, 2023, https://doi.org/10.1093/eurpub/ckad108

In the originally published version of this manuscript, an affiliation was omitted for the second author, Euan Phimister. Professor Phimister’s additional affiliation is:

*Stellenbosch Business School, Stellenbosch University, South Africa*

This error has been corrected online.

