# Supplementary material for: Assessing the impact of energy and fuel poverty on health: a European scoping review
Source: Eur J Public Health. 2023 Jul 12;33(5):764–70. doi: 10.1093/eurpub/ckad108 (PMC10567131; doi:10.1093/eurpub/ckad108)
Supplement: ckad108_Supplementary_Data [file ckad108_supplementary_data.zip › ckad108_Supplementary_Data/ejph-2023-01-om-0004-File003.pdf]

Appendix 1 - Search Strategy

Five search engines (Medline, Embase, Web of Science, Sociological Abstract, and EconLit) were used, in addition to hand searching, relying on the search strategy below:

Concept 1

"Fuel poverty" OR "energy poverty" OR "fuel poor" OR "energy poor" OR "fuel deprivation" OR "energy deprivation" OR "vulnerable energy consumer*" OR "energy insecurity" OR "fuel insecurity" OR "multidimensional energy poverty" OR "cold home*" OR "energy inequality" OR "fuel inequality" OR "heating poverty" OR "heating insecurity" OR "poor heating" OR "energy insufficiency" OR "lack of heating" OR "heating scarcity" OR “warmth deprivation” OR “heating poverty” OR “indoor temperature” OR “household temperature” OR “damp homes” OR “cold housing”

Concept 2

"Health*" OR "Health status disparity" OR "international classification of functioning, disability and health" OR "Non-Communicable Disease*" OR "Communicable disease*" OR "disability" OR "mental health" OR "strength" OR "healthy aging" OR "healthy ageing" OR "chronic disease" OR "chronic illness" OR "disability adjusted life year*" OR “stroke” OR "ADL" OR "daily life activity" OR "ADL disability" OR "IADL" OR "frailty" OR "respiratory infections" OR "respiratory tract infection" OR "common cold" OR "influenza, human" OR "influenza" OR "flu" OR "pneumonia" OR "COVID-19" OR "coronavirus disease 2019" OR "depression" OR "late life depression" OR "depressive disorder" OR "anxiety" OR "anxiety disorder*" OR “cardiac disease” OR “cardiovascular disease” OR “cerebrovascular disorder” OR “heart condition” OR "rheumatic disease" OR "rheumatism" OR "osteoporosis" OR “diabetes” OR “diabetes mellitus” OR “type 2 diabetes” OR "arthritis" OR “injury” OR “injur*” OR “wound” OR "unhealthy" OR “wellbeing” OR “well*being” OR “cold related mortality”

Concept 3

"Europe" OR "Andorra" OR "Austria" OR "Balkan Peninsula" OR "Belgium" OR "Europe, Eastern" OR "Albania" OR "Baltic States" OR "Estonia" OR "Latvia" OR "Lithuania" OR "Bosnia and Herzegovina" OR "Bulgaria" OR "Croatia" OR "Czech Republic" OR "Hungary" OR "Kosovo" OR "Moldova" OR "Montenegro" OR "Poland" OR "Republic of Belarus" OR "Republic of North Macedonia" OR "Romania" OR "Russia" OR "Serbia" OR "Slovakia" OR "Slovenia" OR "Ukraine" OR "European Alpine Region" OR "France" OR "Germany" OR "Gibraltar" OR "Greece" OR "Ireland" OR "Italy" OR "Liechtenstein" OR "Luxembourg" OR "Mediterranean Region" OR "Monaco" OR "Netherlands" OR "Portugal" OR "San Marino" OR "Scandinavian and Nordic Countries" OR "Denmark" OR "Finland" OR "Iceland" OR "Norway" OR "Sweden" OR "Spain" OR "Switzerland" OR "United Kingdom" OR "Channel Islands" OR "England" OR "Northern Ireland" OR "Scotland" OR "Wales" OR "Vatican City" OR "Czechia" OR "Southern Europe" OR "Western Europe"
