# Supplementary material for: Assessing the impact of energy and fuel poverty on health: a European scoping review
Source: Eur J Public Health. 2023 Jul 12;33(5):764–70. doi: 10.1093/eurpub/ckad108 (PMC10567131; doi:10.1093/eurpub/ckad108)
Supplement: ckad108_Supplementary_Data [file ckad108_supplementary_data.zip › ckad108_Supplementary_Data/ejph-2023-01-om-0004-File004.pdf]

Appendix 2

Number of included studies by year

Study Title	Authors	Publication Date	Methodology	Energy and Fuel Poverty Measure	Health Measure
Do fuel-poor households exhibit higher risk factors associated with poor health?	Healy J.	2003	Cross-Sectional	Self-reported inability to adequately heat the home because of low household income and energy inefficient housing	Indirect health measurements: Risk factors associated with ill health
Home is where the hearth is: Grant recipients' views of England's Home Energy Efficiency Scheme (Warm Front)	Gilbertson J, Stevens M, Stiell B, Thorogood N	2006	Qualitative	Eligible participants of the Warm Front Scheme	Self-reported health and wellbeing
Are fuel poverty reduction schemes associated with decreased excess winter mortality in elders? A case study from London, UK	El Ansari W & El-Silimy S	2008	Longitudinal	Fuel expenditure over 10% of total household income	Excess winter mortality
A Randomised Controlled Trial of an Energy Efficiency Intervention for Families Living in Fuel Poverty	Heyman, Bob; Harrington, Barbara; Heyman, Anna	2011	Randomized Control Trial	Spending 7.5-10% of disposable income to achieve room temperatures officially designated as adequate	Self-reported physical health, mental health, and use of health services
Psychosocial routes from housing investment to health: Evidence from England's home energy efficiency scheme	Gilbertson J, Grimsley M, Green G	2012	Cross-Sectional	Eligible participants of the Warm Front Scheme	Self-reported health

Fuel poverty and the health of older people: the role of local climate	de Vries, R; Blane, D	2013	Cross-Sectional	Fuel expenditure over 10% of total household income	Objectively measured respiratory health (peak expiration flow) and hypertension (blood pressure), self-reported depressive symptoms and health
Housing and respiratory health at older ages	Webb, E; Blane, D; de Vries, R	2013	Cross-Sectional	Fuel expenditure over 10% of total household income	Respiratory health measured via a Vitalograph Escort Spirometer
Fuel poverty as a major determinant of perceived health: The case of France	Lacroix E.; Chaton C.	2015	Cross-Sectional	Self-reported feeling cold	Self-reported health
Fuel poverty, affordability, and energy justice in England: Policy insights from the Warm Front Program	Sovacool B K	2015	Other	Eligible participants of the Warm Front Scheme	Not explicit
Fuel poverty in Greece: Quantitative analysis and implications for policy	Atsalis, A; Mirasgedis, S; Tourkolias, C; Diakoulaki, A	2016	Longitudinal	Self-reported inability to keep the home adequately warm	Number of reported Deaths, Cardiovascular Diseases, and Respiratory Infections
Living with fuel poverty in older age: Coping strategies and their problematic implications	Chard, R; Walker, G	2016	Qualitative	Service users of community organisations tackling fuel poverty	Self-reported health and wellbeing

The short-term health and psychosocial impacts of domestic energy efficiency investments in low-income areas: a controlled before and after study	Grey C.N.; Jiang S.; Nascimento C.; Rodgers S.E.; Johnson R.; Lyons R.A.; Poortinga W.	2017	Quasi-Experimental	Communities eligible for the Welsh Government-led energy-efficiency investment programme (Arbed)	Self-reported physical (inclusive of respiratory and asthma symptoms), mental health, and wellbeing
Introduction to a Wales project for evaluating residential retrofit measures and impacts on energy performance, occupant fuel poverty, health and thermal comfort	Littlewood, JR; Karani, G; Atkinson, J; Bolton, D; Geens, AJ; Jahic, D	2017	Other	No explicit EFP measure	No explicit health measure
Housing Policies and Health Inequalities	Mari-Dell'Olmo M.; Novoa A.M.; Camprubi L.; Peralta A.; Vasquez-Vera H.; Bosch J.; Amat J.; Diaz F.; Palencia L.; Mehdipanah R.; Rodriguez-Sanz M.; Malmusi D.; Borrell C.	2017	Cross-Sectional	Self-reported inability to keep the home adequately warm, inability to keep the home adequately warm during wintertime, arrears on utility bills, and inefficient dwelling	Poor self-reported physical and mental health
Documenting Fuel Poverty from the Householders' Perspective	Mould, Ronald; Baker, Keith J	2017	Qualitative	Energy Advocacy Service users	Self-reported physical health, mental health, and wellbeing
Health, Well-Being and Energy Poverty in Europe: A Comparative Study of 32 European Countries.	Thomson, Harriet; Snell, Carolyn; Bouzarovski, Stefan	2017	Cross-Sectional	Self-reported inability to heat the home adequately because of low household income	Self-reported health and wellbeing

Energy justice, unequal access to affordable warmth, and capability deprivation: A quantitative analysis for Belgium	Bardaux, F; Vandeschrick, C; Moezzi, M; Frogneux, N	2018	Cross-Sectional	Self-reported inability keep the home adequately warm, arrears on utility bills, and inefficient dwelling	Self-reported bodily health and emotions
Housing conditions and non-communicable diseases among older adults in Ireland	Gibney, S; Ward, M; Shannon, S	2018	Cross-Sectional	Self-reported inability to keep the household adequately warm	Self-reported health status, respiratory health problems and bone and joint conditions
Objective vs. Subjective Fuel Poverty and Self-Assessed Health	Llorca, Manuel; Rodriguez-Alvarez, Ana; Jamasb, Tooraj	2018	Longitudinal	Self-reported inability to keep their home adequately warm and the Minimum Income Standard	Self-assessed health
Variation in cold-related mortality in England since the introduction of the cold weather plan: Which areas have the greatest unmet needs?	Murage P.; Hajat S.; Bone A.	2018	Ecological	Area level EFP vis households whose fuel costs are above the national median, and whose residual income would fall below the poverty line after paying for their fuel consumption	Mortality, cardiac mortality, and respiratory mortality
The Impact of Fuel Poverty Upon Self-Reported Health Status among the Low-Income Population in Europe	Bosch, Jordi; Palencia, Laia; Malmusi, Davide; Mari-Dell'Olmo, Marc; Borrell, Carme	2019	Cross-Sectional	Self-reported inability to keep the home adequately warm, arrears on utility bills, dwelling not comfortably warm during the winter and inefficient dwelling	Self-reported health
Energy Poverty and Life Satisfaction: Structural Mechanisms and Their Implications	Druica, E; Goschin, Z; Ianole-Calin, R	2019	Cross-Sectional	Self-reported unaffordability to adequate heat one's dwelling	Self-assessed satisfaction with life and health status
Use of Simple Telemetry to Reduce the Health Impacts of Fuel Poverty and Living in Cold Homes	Pollard, A; Jones, T; Sherratt, S; Sharpe, RA	2019	Cross-Sectional	Self-reported inability to keep the household adequately warm	Self-reported physical health, mental health, and wellbeing

Energy poverty and gender in England: A spatial perspective	Robinson C	2019	Spatial Analysis	Unaffordability of energy, energy inefficiency, inflexibility in the built environment including tenure arrangements, and energy-related needs and practices that increase energy use	Exposure to physiological and mental health impacts
A novel Index of Vulnerable Homes: Findings from application in Spain	Castano-Rosa R.; Solis-Guzman J.; Marrero M.	2020	Cross-Sectional	EFP index based on monetary cost, energy, and thermal comfort	Quality-adjusted life years score based on self-perceived mobility, ability to perform self-care activities, ability to perform usual activities, pain/discomfort, anxiety/depression
Energy poverty goes south? Understanding the costs of energy poverty with the index of vulnerable homes in Spain	Castano-Rosa, R, Solis-Guzman, J & Marrero M	2020	Qualitative	EFP index based on monetary cost, energy, and thermal comfort	Health -Related Quality-Life Costs
The association of energy poverty with health, health care utilisation and medication use in southern Europe	Oliveras L.; Artazcoz L.; Borrell C.; Palencia L.; Lopez M.J.; Gotsens M.; Peralta A.; Mari-Dell'Olmo M.	2020	Cross-sectional	Self-reported inability to maintain the dwelling at an adequate temperature during the cold and/or warm months	Self-reported health, mental health via the General Health Questionnaire, quality of life via Euroqol 5D-5L, self-reported chronic morbidity, overweight, domestic injuries, health services use, and medication use
Energy Poverty as a Restriction of Multiple Capabilities: A Systemic Approach for Belgium	Bartiaux, Francoise; Day, Rosie; Lahaye, Willy	2021	Qualitative	Individuals with delays in paying an energy bill and insufficient financial means	Self-perceived bodily health and emotions

Energy poverty, its intensity and health in vulnerable populations in a Southern European city	Carrere, J; Peralta, A; Oliveras, L; Lopez, MJ; Mari-Dell'Olmo, M; Benach, J; Novoa, AM	2021	Cross-Sectional	Self-reported inability to keep the home warm, inability to pay utility bills on time, and presence of dampness and rot in the dwelling	Self-perceived poor health, asthma, chronic bronchitis, and depression and/or anxiety
Getting Warmer: Fuel Poverty, Objective and Subjective`Health and Well-Being*	Davillas A., Burlinson A., Liu H. H.	2021	Cross-Sectional	Self-reported inability to maintain household warmth and low-income-high-cost indicator	Self-reported health and life-satisfaction and biomarker data
The impact of household energy poverty on the mental health of parents of young children	Mohan G.	2021	Cohort Study	Self-reported inability to keep the household adequately warm, needing to go without heating in the last 12months due to a lack of money, and arrears on utility bills	Self-reported parental depression
The association of energy poverty with health and wellbeing in children in a Mediterranean city	Oliveras L.; Borrell C.; Gonzalez-Pijuan I.; Gotsens M.; Lopez M.J.; Palencia L.; Artazcoz L.; Mari-Dell'olmo M.	2021	Cross-Sectional	Self-reported inability to maintain the dwelling at an adequate temperature during the cold and/or warm months	Caregiver reported child health, mental health via the Strengths and Difficulties Questionnaire, health-related quality of life through the Kidscreen-10, asthma through a chronic morbidity checklist, and overweight via BMI
Young, Poor, and Sick: The Public Health Threat of Energy Poverty for Children in Ireland	Mohan, Gretta	2021	Cohort Study	Self-reported inability to keep the household adequately warm, and needing to go without heating in the last 12months due to a lack of money	Caregiver reports of child respiratory disease

“It’s changed my life not to have the continual worry of being warm” – health and wellbeing impacts of a local fuel poverty programme: a mixed-methods evaluation	Sawyer A, Sherriff N, Bishop D, Darking M, and Huber J. W.	2022	Mixed-Methods	Beneficiaries of the Healthy Homes programme	Self-reported health, wellbeing via the Warwick-Edinburgh Mental Wellbeing Scale
Combining self-reported and sensor data to explore the relationship between fuel poverty and health well-being in UK social housing	Tu G, Morrissey K, Sharpe R. A, & Taylor T	2022	Cross-Sectional	Self-reported inability to keep the household adequately heated, being able to afford energy needed, and being able to prevent the home from housing faults due to poor insulation, an inability to maintain a 18C warmth standard, and an inability to maintain a 17C warmth standard for healthy adults	Physical health, mental health, and wellbeing was measures via the SF-12TM version 2
