# Supplementary material for: Assessing the impact of energy and fuel poverty on health: a European scoping review
Source: Eur J Public Health. 2023 Jul 12;33(5):764–70. doi: 10.1093/eurpub/ckad108 (PMC10567131; doi:10.1093/eurpub/ckad108)
Supplement: ckad108_Supplementary_Data [file ckad108_supplementary_data.zip › ckad108_Supplementary_Data/ejph-2023-01-om-0004-File005.pdf]

Appendix 3 - Additional References

41. Druică E, Goschin Z, Ianole-Călin R. Energy poverty and life satisfaction: Structural mechanisms and their implications. Energies (Basel). 2019;12(20).

42. Grey CNB, Jiang S, Nascimento C, Rodgers SE, Johnson R, Lyons RA, et al. The short-term health and psychosocial impacts of domestic energy efficiency investments in low-income areas: a controlled before and after study. BMC Public Health. 2017;17(1).

43. Gibney S, Ward M, Shannon S. Housing conditions and non-communicable diseases among older adults in Ireland. Qual Ageing Older Adults. 2018;19(3).

44. Murage P, Hajat S, Bone A. Variation in cold-related mortality in England since the introduction of the cold weather plan: Which areas have the greatest unmet needs? Int J Environ Res Public Health. 2018;15(11).

45. Robinson C. Energy poverty and gender in England: A spatial perspective. Geoforum. 2019;104.

46. Boardman B. Fuel Poverty: From Cold Homes to Affordable Warmth. London, UK: Belhaven Press; 1991.

47. Robine JM, Cheung SLK, le Roy S, van Oyen H, Griffiths C, Michel JP, et al. Death toll exceeded 70,000 in Europe during the summer of 2003. C R Biol. 2008;331(2).

48. De Vries R, Blane D. Fuel poverty and the health of older people: The role of local climate. Journal of Public Health (United Kingdom). 2013;35(3).

49. Sovacool BK. Fuel poverty, affordability, and energy justice in England: Policy insights from the Warm Front Program. Energy. 2015;93.

50. Alkire S, Foster J. Understandings and misunderstandings of multidimensional poverty measurement. J Econ Inequal. 2011;9(2).

51. Gilbertson J, Grimsley M, Green G. Psychosocial routes from housing investment to health: Evidence from England's home energy efficiency scheme. Energy Policy. 2012;49.

52. Eurostats: statistics explained. Glossary: Material Deprivation. European Union. 2023.

53. Beatty TKM, Blow L, Crossley TF. Is there a 'heat-or-eat' trade-off in the UK? J R Stat Soc Ser A Stat Soc. 2014;177(1).

54. Stronks K, Van de Mheen H, Van den Bos J, Mackenbach JP. The interrelationship between income, health and employment status. Int J Epidemiol. 1997;26(3).

55. Ecob R, Davey Smith G. Income and health: What is the nature of the relationship? Soc Sci Med. 1999;48(5).

56. World Health Organization (WHO). WHO Housing and health guidelines, Recommendations to promote healthy housing for a sustainable and equitable future. Department of Housing and Urban Development. 2018.

57. Healy J. Do fuel-poor households exhibit higher risk factors associated with poor health? Int J Health Promot Educ. 2003;41(1).

58. Mark Olden, Martin Pigeon. Europe's Dark Winter How will people and forests survive the energy crisis? [Internet]. 2022. Available from: www.fern.org

59. Alice Taylor, Kira Taylor, Krassen Nikolov, Pekka Vanttinen, Vlad Makszimov. Firewood prices, shortages spell cold winter for Europe’s poorest. EURACTIV. 2022 Sep 7.

60. Marton Dunai, Raphael Minder. ‘People will burn anything’: energy poverty and pollution hit eastern Europe. Financial Times. 2022 Oct.

61. Littlewood JR, Karani G, Atkinson J, Bolton D, Geens AJ, Jahic D. Introduction to a Wales project for evaluating residential retrofit measures and impacts on energy performance, occupant fuel poverty, health and thermal comfort. In: Energy Procedia. 2017.

62. Llorca M, Rodriguez-Alvarez A, Jamasb T. Objective vs. subjective fuel poverty and self-assessed health. Energy Econ. 2020;87.

63. Mohan G. The impact of household energy poverty on the mental health of parents of young children. Journal of Public Health (United Kingdom). 2022;44(1).
